# Exploring Some Aspects Associated with Dentine Hypersensitivity in Children

**DOI:** 10.1155/2015/764905

**Published:** 2015-03-24

**Authors:** Caleb Shitsuka, Fausto Medeiros Mendes, Maria Salete Nahás Pires Corrêa, Mariana Ferreira Leite

**Affiliations:** ^1^University of São Paulo, São Paulo, SP, Brazil; ^2^University of Cruzeiro do Sul, São Paulo, SP, Brazil

## Abstract

*Background*. The etiology of dentine hypersensitivity (DH) is still inconclusive and there are few studies concerning it in children. *Aim*. To evaluate clinical, dietary, and salivary variables in children with DH complaints. *Design*. Forty-eight children were asked about DH. Data regarding dietary habits were collected from the children's parents and an examination was performed to determine dental erosion. Dental biofilm was estimated by oral hygiene status, according to Greene and Vermillion's Simplified Oral Hygiene Index (OHI-S). Whole saliva was collected under mechanical stimulation and evaluated salivary flow rate, initial pH, buffer capacity, and calcium and phosphate concentrations. The temperature of soft drinks, drinking method, sense of bitter taste, and other variables were also determined. Possible factors associated with DH were analyzed by univariate and multiple Poisson regression analyses. The prevalence ratio (PR) values and 95% confidence intervals (95% CI) were calculated. *Results*. DH was associated with the presence of dental erosion (PR; 95% CI = 2.23; 1.05 to 4.71) and salivary flow rate (2.49; 1.05 to 5.91). When the presence of erosion was not included, other variables were retained as follows: bitter taste (2.36; 1.38 to 4.03), OHI-S (0.47; 0.23 to 0.97). *Conclusion*. DH in children is associated with factors related to dental erosion.

## 1. Introduction

Dentine hypersensitivity (DH) is one of the most frequent complaints in the dental office related to loss of tooth structure [1–3]. It is characterized by acute pain of sudden onset and short duration that is typically caused by thermal, evaporative, tactile, osmotic, or chemical stimulation of open dentine tubules and exposed surface and cannot be ascribed to any other form of defect or pathology [4]. The “hydrodynamic theory” proposed by Brännström et al. [5] is widely used to explain the mechanism of action of DH; however, its etiologic factors and diagnosis remain inconclusive [6].

The prevalence for HD is not well understood; however, some studies indicate that about 30% of the general population report HD [7–9]. Some of the main etiological factors of DH are chronic trauma from toothbrushing, acid erosion (e.g., as a result of environmental exposure, gastric regurgitation, or dietary substances), and anatomical factors [10].

Saliva plays a key role in maintaining the mineralized structure of the tooth due to its participation in the pellicle formation, buffer capacity, and supersaturation of electrolytes [11]. However, the association of salivary parameters with DH has not been extensively studied. Furthermore, since protection of the tooth surface is of fundamental importance in the prevention and treatment of DH, we believe it is of interest to study the role of dental biofilm in individuals who present this symptom. Despite the possibility of aciduric microorganism colonization, under healthy conditions, dental biofilm has a protective function in relation to the enamel surface, acting as selective barrier against acid penetration and as a reserve of ions, such as calcium fluoride [12]. A negative correlation between plaque accumulation and the presence of DH has been demonstrated, suggesting that dental biofilm could be a protective factor for the dental surface [13]. However, few studies address this possible association between the presence of biofilm and the reduction in DH [14].

Although several studies related to the prevalence, etiology, and treatment of DH have been published, most of them have focused on its occurrence in adolescents and adults. In children, DH has been related to dental developmental defects, such as amelogenesis imperfecta [15] and hypomineralization [16]. There is a lack of studies concerning DH in children with no developmental dental defects. Thus, the aim of this study was to evaluate clinical and salivary aspects associated with DH in children. This is the first study that investigated associated clinical and salivary factors with DH in children.

## 2. Material and Methods

### 2.1. Subjects

This cross-sectional study was approved by the Human Research Ethics Committee of the Cruzeiro do Sul University under protocol number 015/2010. After being informed of the purpose of the investigation, written informed consent for participation and publication was obtained from the adult responsible for each child who agreed to participate in this study.

A group of 48 male and female children (aged 4–9 years old) attending the Clinic of Pediatric Dentistry of Cruzeiro do Sul University was enrolled in this study. A convenience selection procedure was performed according to the presence of dental erosion. Children were initially selected according to the presence of dental erosion, after which control children with no dental erosion were selected and paired for age and sex. Additional inclusion criteria were children with no caries or with absence of systemic disease. Children exhibiting incipient caries, failed restorations, cracks or dental fractures, and teeth with reversible or irreversible inflammatory processes of the pulp were excluded.

### 2.2. Collection of the Independent Variables

The patients were examined according to dental erosion criteria and oral hygiene status by a single examiner in a dental chair condition for a good evaluation.

Oral hygiene was assessed according to Greene and Vermillion's Simplified Oral Hygiene Index (OHI-S) [17]. The dental surfaces were stained using 3% fuchsin and the dental surfaces of some teeth were classified according to the amount of dental biofilm. The children were asked to maintain fasting and avoid oral hygiene for a minimum of 2 h prior to the examination.

After dental cleaning with rotating bristle brush and pumice/water slurry, the teeth were assessed regarding dental erosion. We used the O'Brien index [18] to assess the presence of dental erosion. In addition, the children were asked whether they have experienced a bitter taste in their mouth.

A face-to-face interview was performed with the caregivers, who answered a questionnaire concerning their children's dietary habits. The questionnaire detailed the consumption and frequency of foods considered erosive, such as citrus fruits, fruit juices, carbonated soft drinks, tea, and acidic candies. The parents were also asked about certain habits concerning soft drink consumption, such as the temperature of the beverage and drinking methods (use of a straw, swallowing immediately, or holding the beverage in the mouth before swallowing).

### 2.3. Diagnosis of DH

The presence of dentine hypersensitivity was determined by a single examiner trained to diagnose the problem, according to the criteria proposed by Porto et al. [6]. The examiner performed a detailed clinical history and dental examination in order to eliminate possible causes of pain, which permitted the differentiation of dentine hypersensitivity from other dental pathologies. The air jet from a triple syringe was used on surfaces of the teeth to provoke a response from the patient and identify areas with suspected dentin hypersensitivity [6].

### 2.4. Salivary Collection

At least 2 h after the previous meal and after being oriented not to brush the teeth, stimulated whole saliva with Parafilm was collected between 8 and 10 a.m., to minimize the circadian rhythm effects. Saliva produced in the first 10 s was discarded and the subsequent saliva was collected for exactly 5 min in a graduated cylinder to calculate the initial flow rate (mL/min). During the collection period, all individuals remained comfortably seated in a ventilated and illuminated room/environment. Soon after collection, a part of salivary sample was frozen in dry ice, transported to the laboratory, and stored at −80°C until analysis.

Immediately after saliva collection, both initial pH and buffer capacity were determined using a portable pH meter (Digimed DU-2, Sao Paulo, Brazil). The salivary buffer capacity of whole saliva was determined by acid titration of 1 mL of saliva with a constant amount of 0.01 N HCl (0.2 mL). After each addition of acid, the change in pH was monitored until the saliva-acid solution reached pH 5.0. For interpretation of the results regarding pH changes, the buffer capacity of whole saliva was analyzed using pH intervals (initial pH-7.0; pH 6.9–6.0; pH 5.9–5.0). For practical purposes, the salivary buffer capacity was expressed as the volume (mL) of the acid added to 1 mL of saliva.

### 2.5. Biochemical Analysis

Calcium concentration was performed by method described by Nogueira et al. [19]. Salivary calcium is precipitated by chloranilic acid, in the form of calcium chloranilate. After washing in isopropyl alcohol 50%, the precipitate is dissolved in an aqueous solution of 5% EDTA (ethylenediaminetetraacetic acid). Calcium phosphate (0.1 mg/mL) is used as a standard solution and the result is determined by reading in a spectrophotometer at 520 nm.

The concentration of phosphorus in the saliva was determined by the modified method of Fiske and Subbarow [20]. Saliva is deproteinized by incubation with TCA (trichloroacetic acid) 1.2 M. Once deproteinized, the phosphate minerals react with molybdic acid (solution of 2.5% ammonium molybdate in 10 N sulfuric acid) to form a complex of phosphomolybdic acid, which is then reduced by ascorbic acid to form a blue colored complex in which the color intensity is proportional to the amount of inorganic phosphorus. The standard curve is determined by reaction with a 1 Mmol/mL standard phosphorus solution and by spectrophotometry at 720 nm.

### 2.6. Statistical Analyses

For the analysis, each child was the experimental unit and DH was considered the outcome. The independent variables were as follows: sex, male or female; age, a continuous variable in years; dental erosion, absent or present; bitter taste, no or yes; temperature of soft drink, room temperature versus cold or iced drink; usual drinking method, by swallowing immediately or using straw versus holding the beverage in the mouth before swallowing; and OHI-S, a continuous variable. The independent variables related to dietary habits consumption were as follows: soft drinks intake, some days versus every day; juice intake, 3 times per day or less versus more than 3 times/day; tea intake, never or rarely versus once a week or more; citrus fruits intake, never or rarely versus once a week or more; and acidic candies intake, never or rarely versus once a week or more. The salivary parameters investigated were salivary flow rate, calcium and phosphorus concentration, initial pH, and buffer capacity (in each pH range and total).

The analyses were performed through Poisson regression with robust variance. First, univariate analyses were performed with each independent variable alone. The prevalence ratio (PR) values and 95% confidence intervals (95% CI) were calculated. The variables which achieved a *P* value < 0.20 in the univariate analyses were tested in the multiple model using a forward stepwise procedure. Following multiple Poisson regression analysis, only variables with a *P* value < 0.05 were maintained in the final model.

## 3. Results

A total of 48 children participated in this study and of these, 20 (41%) presented DH and 28 (59%) presented no DH. The mean (standard deviation) age of the DH group was 6.72 (1.64) years old and the control group (with no DH) was 6.93 (1.61) years old. The sample comprised 26 males (54.2%) and 22 females (45.8%).

In the univariate analysis, we observed that children presenting signs of dental erosion showed higher prevalence of DH than children with no dental erosion. Children that reported a bitter taste also presented higher prevalence of DH (Table 1). Concerning dietary habits, no dietary habit was significantly associated with higher prevalence of DH (Table 2).

Considering the salivary parameters, children presenting higher salivary flow rate exhibited higher prevalence of DH. Other salivary parameters, however, showed no significant association with DH (Table 3).

In the multiple Poisson regression analysis, we obtained a multiple model containing two variables as follows: salivary flow rate and presence of dental erosion. In this model, children exhibiting dental erosion presented twice the DH, and children with higher salivary flow rate also presented higher prevalence of DH (Table 4, model 1). When we tested other variables, such as bitter taste and OHI-S together with dental erosion, most of the variables lost their significance. Thus, we decided to construct other multiple models without dental erosion. In the second multiple regression model, we observed that children with bitter taste in their mouth and with higher salivary flow rate presented higher prevalence of DH. In contrast, children exhibiting higher levels of OHI-S presented lower prevalence of DH (Table 4, final model 2).

## 4. Discussion

Knowledge of etiologic factors of a disease or symptoms is of fundamental importance for the definition of preventive and therapeutic measures. This study evaluated clinical and salivary factors that could be involved in the etiology of DH in children. This condition has not been extensively studied in children, and most studies have reported association with developmental defects of enamel [15, 16]. In our study, we observed association with factors related to dental erosion. Furthermore, we observed that children with DH presented an increased salivary flow rate in stimulated whole saliva compared with children without any complaints. To our knowledge, this is the first study that has investigated the association of salivary parameters with DH in children.

The most important finding of our study is that higher prevalence of DH was determined in children with factors related to dental erosion. Some similarity exists between the etiology of dental erosion and DH [21]. Dentine hypersensitivity is associated with dentine exposed to the oral environment while dental erosion was defined by Pindborg [22] as the superficial loss of hard tissues of the teeth by a chemical process that does not involve bacterial action. The action of common etiologic factors can explain this association.

When we included dental erosion in the final multiple model, a sense of bitter taste and the presence of dental biofilm lost their significance. The loss of significance of dental biofilm could be due to colinearity with dental erosion, since some authors have reported a protective effect of dental biofilm against dental erosion [23]. Furthermore, acids responsible for the etiology of dental erosion can be of intrinsic origin, which includes gastrointestinal disorders, gastroesophageal disease, anorexia, and bulimia, in which regurgitation and frequent vomiting are common [24]. Although the investigation of intrinsic factors of erosive lesions was not the focus of our study, the children reported frequently sensing a bitter taste, which was associated with DH. The bitter taste could be indicative of the participation of intrinsic factors in DH, a fact that requires corroboration by future studies.

Another interesting finding was the significant negative association between dental biofilm and DH. This finding is in agreement with previous studies that showed the protective effect of dental biofilm in cases of DH [13, 23]. The acquired enamel pellicle and dental biofilm are a natural protection of mineralized surface against erosion or abrasion, preventing the output of calcium and phosphate from the liquid phase of hydroxyapatite and direct contact by acid on the tooth surface, acting as a selective barrier of ions [25]. Stimulation of natural defense factors could have preventive and therapeutic potential in the treatment of DH and could prove to be a subject of interest in medical science, considering that the occlusion of dentinal tubules and surface protection are one of the first approaches in the treatment of DH resulting in sealed tubules and isolation from external stimuli [6, 26–28].

Confirmation of the association between factors related to dental erosion and DH was obtained in the alternative final multiple model that we constructed. Following the exclusion of the presence of dental erosion, both bitter taste and OHI-S variables were significantly associated with DH in the final model.

Lifestyle has changed over the years and the current consumption of acidic foods and beverages is extremely high, especially among children [24, 29, 30]. The acidic components are common in modern eating habits, particularly from fruits [31]. Chemical stimulus is one of the factors responsible for dentine hypersensitivity, which generates great physical and psychological discomfort for patients [6]. Although an acidic diet has been related to DH in some studies [30, 32–34], our results showed no association between DH and the frequency or manner of intake of erosive foods and drinks.

Although saliva plays an essential role in maintaining the integrity of mineralized tissues of the tooth, no previous reports have considered their importance in cases of DH. The only salivary factor positively associated with DH was the salivary flow rate. The stimulation of salivary flow can enhance the defense functions performed by saliva, such as mechanical cleaning, antimicrobial properties, mucosal lubrication, clearance of food residues, and buffer capacity [11]. It is possible that the salivary flow rate could be stimulated by the same factor that causes the bitter taste often reported by the children with DH. For this reason, it would be interesting to develop studies that address gastrointestinal problems or intrinsic factors of dental erosion and their possible correlations with DH.

Despite its important contribution concerning the clinical and salivary elements on the etiology of DH, some limitations to the study should be mentioned. Diagnosis of DH in individuals in the age range of group studied should be conducted very carefully, because children can show difficulty in verbalizing and can be induced to error of complaint by suggestion of pain. Furthermore, we selected a convenience sample with children presenting dental erosion and counterparts without erosion. The small sample size is also a limiting factor. Notwithstanding these limitations, this pioneer study opens new avenues that should assist in improving current understanding concerning DH in children.

## 5. Conclusion

In conclusion, increased salivary flow rate and factors related to the presence of dental erosion are associated with the occurrence of DH in children.

This study provides important elements that will help the pediatric dentist to understand the DH in children. Furthermore, these results may be an alert because probably intrinsic factors of dental erosion which cause the bitter taste and salivary stimulation are associated with DH in children. The intrinsic factors of dental erosion need to be carefully investigated.

## Figures and Tables

**Table 1:** Univariate Poisson regression analysis of possible factors related to the child associated with dental hypersensitivity.

Independent variables	Total *N*	Children with DH *N* (%)	PR (95% CI)	*P* ^*^
Age (cont.)			0.97 (0.78 to 1.19)	0.784
Sex				
(ref.: male)	26	11 (42.3)	1.00	0.923
Female	22	9 (40.9)	0.97 (0.49 to 1.91)	
Dental erosion				
(ref.: absent)	24	6 (25.0)	1.00	0.033
Present	24	14 (58.3)	2.33 (1.07 to 5.09)	
Bitter taste				
(ref.: no)	39	13 (33.3)	1.00	0.004
Yes	9	7 (77.8)	2.33 (1.32 to 4.13)	
Temperature of soft drink				
(ref.: room temperature)	19	9 (47.4)	1.00	0.517
Cold/iced	29	11 (37.9)	0.80 (0.41 to 1.57)	
Usual drinking method				
(ref.: Swallowing immediately)	39	14 (35.9)	1.00	0.054
Holding the beverage in the mouth before swallowing	9	6 (66.7)	1.86 (0.99 to 3.49)	
Simplified Oral Hygiene Index (cont.)			0.47 (0.24 to 0.94)	0.033

DH = dental hypersensitivity; PR = prevalence ratio; 95% CI = 95% confidence interval.

^*^Calculated by the Wald test.

**Table 2:** Univariate Poisson regression analysis of the association between children's dietary habits and presence of dental hypersensitivity.

Dietary habits variables	Total *N*	Children with DH *N* (%)	PR (95% CI)	*P* ^*^
Soft drinks intake				
(ref.: some days)	27	9 (33.3)	1.00	0.192
Everyday	21	11 (52.4)	1.57 (0.79 to 3.10)	
Juice intake				
(ref.: 3 times/day or less)	24	9 (37.5)	1.00	0.564
More than 3 times/day	24	11 (45.8)	1.22 (0.62 to 2.42)	
Tea intake				
(ref.: never/rarely)	35	13 (37.1)	1.00	0.277
Once a week or more	13	7 (53.8)	1.45 (0.74 to 2.83)	
Citrus fruits intake				
(ref.: never/rarely)	32	13 (40.6)	1.00	0.836
Once a week or more	16	7 (43.8)	1.08 (0.53 to 2.18)	
Acidic candies intake				
(ref.: never/rarely)	8	1 (12.5)	1.00	0.164
Once a week or more	40	19 (47.5)	3.80 (0.58 to 24.95)	

DH = dental hypersensitivity; PR = prevalence ratio; 95% CI = 95% confidence interval.

^*^Calculated by the Wald test.

**Table 3:** Univariate Poisson regression analysis between salivary parameters and presence of dental hypersensitivity.

Salivary parameters	Without DH mean (SD)	With DH mean (SD)	PR (95% CI)	*P* ^*^
Salivary flow rate (mL/min)	0.49 (0.27)	0.66 (0.31)	2.56 (1.28 to 5.12)	0.008
Calcium concentration (*μ*g/mL)	194.9 (61.6)	182.1 (40.8)	1.00 (0.99 to 1.01)	0.404
Phosphate concentration (mg/mL)	1.16 (0.33)	1.16 (0.25)	1.01 (0.33 to 3.07)	0.988
Initial pH	7.42 (0.28)	7.45 (0.36)	1.23 (0.39 to 3.89)	0.725
Total buffer capacity (mL HCl 0.01 N)	1.31 (0.31)	1.33 (0.39)	1.08 (0.39 to 3.03)	0.879
Buffer capacity in each pH range (mL HCl 0.01 N)				
pHi-7.0	0.34 (0.24)	0.32 (0.24)	0.84 (0.18 to 3.87)	0.827
6.9–6.0	0.60 (0.19)	0.63 (0.27)	1.42 (0.31 to 6.52)	0.836
5.9–5.0	0.38 (0.10)	0.38 (0.09)	1.10 (0.03 to 38.86)	0.958

DH = dental hypersensitivity; PR = prevalence ratio; 95% CI = 95% confidence interval; SD = standard deviation.

^*^Calculated by the Wald test.

**Table 4:** Multiple Poisson regression analysis between independent variables and presence of dental hypersensitivity.

Salivary parameters	Adjusted prevalence ratio (95% confidence interval)	*P* ^*^
Final multiple model 1		0.021
Salivary flow (mL/min)	2.49 (1.05 to 5.91)	
Presence of dental erosion	2.23 (1.05 to 4.71)	
Final multiple model 2		<0.001
Salivary flow (mL/min)	2.88 (1.58 to 5.25)	
Bitter taste	2.36 (1.38 to 4.02)	
Simplified Oral Hygiene Index	0.47 (0.23 to 0.97)	

^*^ *P* value of the final model.
